# TGF-β Activated Kinase 1 (TAK1) Is Activated in Microglia After Experimental Epilepsy and Contributes to Epileptogenesis

**DOI:** 10.1007/s12035-023-03290-2

**Published:** 2023-03-02

**Authors:** Dilaware Khan, Peter Bedner, Julia Müller, Fabienne Lülsberg, Lukas Henning, Marco Prinz, Christian Steinhäuser, Sajjad Muhammad

**Affiliations:** 1 Department of Neurosurgery, Medical Faculty and University Hospital Düsseldorf, Heinrich-Heine-University, Düsseldorf, Germany; 2 Institute of Cellular Neurosciences, Medical Faculty, University of Bonn, Venusberg Campus 1, 53127 Bonn, Germany; 3 Institute of Neuropathology, Faculty of Medicine, University of Freiburg, Freiburg, Germany; 4 Center for Basics in NeuroModulation (NeuroModulBasics), Faculty of Medicine, University of Freiburg, Freiburg, Germany; 5 Signalling Research Centres BIOSS and CIBSS, University of Freiburg, Freiburg, Germany; 6 Department of Neurosurgery, University of Helsinki and Helsinki University Hospital, Helsinki, Finland

**Keywords:** Inflammation, Epilepsy, TAK1, Microglia

## Abstract

Increasing evidence suggests that inflammation promotes epileptogenesis. TAK1 is a central enzyme in the upstream pathway of NF-κB and is known to play a central role in promoting neuroinflammation in neurodegenerative diseases. Here, we investigated the cellular role of TAK1 in experimental epilepsy. C57Bl6 and transgenic mice with inducible and microglia-specific deletion of Tak1 (Cx3cr1^CreER^:Tak1^fl/fl^) were subjected to the unilateral intracortical kainate mouse model of temporal lobe epilepsy (TLE). Immunohistochemical staining was performed to quantify different cell populations. The epileptic activity was monitored by continuous telemetric electroencephalogram (EEG) recordings over a period of 4 weeks. The results show that TAK1 was activated predominantly in microglia at an early stage of kainate-induced epileptogenesis. Tak1 deletion in microglia resulted in reduced hippocampal reactive microgliosis and a significant decrease in chronic epileptic activity. Overall, our data suggest that TAK1-dependent microglial activation contributes to the pathogenesis of chronic epilepsy.

**Supplementary Information:**

The online version contains supplementary material available at 10.1007/s12035-023-03290-2.

## Background

Epilepsy is one of the most common neurological disorders and affects approximately 50 million individuals worldwide. Despite the successful development of a wide range of antiepileptic drugs, one-third of epilepsies are resistant to drug treatment [1]. The reason for drug resistance might be the lack of mechanistic approaches during drug development, as currently available drugs mainly treat epilepsy symptomatically via blockage of neuronal excitability and do not disrupt the pathophysiological mechanisms underlying the disease process. There is ample evidence that inflammatory processes within the brain constitute a common and crucial mechanism in the pathophysiology of epilepsy [2–6]. Activated microglia and astrocytes have been observed in tissue resected from patients with intractable epilepsy [7, 8] and have also been shown in animal models of epilepsy [9–12]. In addition to local brain inflammation, an increasing body of evidence has demonstrated that blood-borne inflammatory cells infiltrate into the epileptic lesion and contribute to the pathophysiology of epilepsy through inflammatory mediators [13].

Inflammatory damage-associated molecular patterns and cytokines such as HMGB1, IL-1β, and TNF-α either from local or infiltrated cells seem to play an important role during ictogenesis [10, 14]. The downstream pathway of these proinflammatory cytokines converges at the level of the MAP kinase TAK-1, which is a key enzyme for the phosphorylation of MAP2Ks (including MKK 4/7, MKK 3/6, and IKK) that subsequently leads to the activation of downstream signals, such as NF-κB, JNK, and p38 MAPK. The transcription factor NF-κB is a key regulator in the synthesis of cytokines (such as IL-1 β, TNF-α, and IL-6), chemokines, and receptors (including TLRs and IL-1R).

The NF-κB pathway modulates a variety of genes that contribute to cell death and survival and importantly to synaptic molecular reorganization and plasticity [15, 16]. Cell death and synaptic reorganization are key processes that occur during ictogenesis. Another downstream kinase, p38 MAPK, is a strong modulator of the biophysical properties of h-channels. Altered h-channels potentially play a role in the pathophysiology of seizures [17, 18].

Both upstream and downstream molecules of TAK1 are strongly linked to the pathophysiology of seizure; they are either involved in causing the seizure or they are a consequence of the seizure. Using the intracortical kainate mouse model of epilepsy, here we show that TAK1 is activated predominantly in microglia during epilepsy and genetic deletion of TAK1 in microglial cells reduced chronic epileptic activity in experimental epilepsy.

## Methods

### Experimental Animals

Both male and female Cx3cr1^CreER^:Tak1^fl/fl^ and Tak1^fl/fl^ animals from C57/Bl6 background were used for experiments. In these mice, TAK1 is conditionally knocked out by Cre-recombinase fused to a mutant estrogen ligand-binding domain (Cx3cr1^CreER^) [19]. To induce recombination, mice received intraperitoneal (i.p.) injections of tamoxifen (TAM, Sigma-Aldrich, Steinheim, Germany) dissolved in sunflower seed oil (Sigma-Aldrich) and ethanol at a ratio of 1:10. TAM (1 mg/mouse) was injected twice daily for 5 consecutive days 6 or 7 weeks after birth. The animals were used for experiments 3 weeks after TAM injection. For genotyping, genomic DNA was obtained from small tail tips of 3-week-old mice and primers were used according to a previously published method [20].

### Temporal Lobe Epilepsy Model and EEG Analysis

We employed the unilateral intracortical kainate mouse model of TLE as described previously [21]. Briefly, mice were anesthetized by i.p. injection of a mixture of medetomidine (Cepetor, CP-Pharma, Burgdorf, Germany, 0.3 mg/kg, i.p.) and ketamine (Ketamidor, WDT, Garbsen, Germany, 40 mg/kg, i.p.) and placed in a stereotaxic frame equipped with a manual microinjection unit (TSE Systems GmbH, Bad Homburg, Germany). Two 0.7-mm diameter holes were drilled bilaterally 1.5 mm from the sagittal suture and 2 mm posterior to the bregma. A total volume of 70 nl of a 20-mM solution of kainate (Tocris, Bristol, UK) in 0.9% sterile NaCl was stereotactically injected into the neocortex just above the right dorsal hippocampus (1.7 mm from scull surface). Electrographic seizures were detected via skull surface electrodes implanted directly after kainate injection. Telemetric transmitters (TA10EA-F20, TA11ETAF10; Data Sciences International, St. Paul, MN) were placed subcutaneously into the right abdominal region and the two monopolar leads were connected to stainless steel screws (length 2 mm; thread diameter 0.8 mm) inserted into the drilled holes. The attached leads were covered with dental cement, skin incisions sutured, and anesthesia stopped with atipamezole (Antisedan, Orion Pharma, Hamburg, Germany, 300 mg/kg, i.p.). To reduce pain, mice were subsequently injected for 3 days with carprofen (Rimadyl, Pfizer, Karlsruhe, Germany). Furthermore, 0.25% Enrofloxacin (Baytril, Bayer, Leverkusen, Germany) was administered via drinking water to reduce the risk of infection. Mice were then returned to clean cages and placed on individual radio receiving plates (RPC-1; Data Sciences International), which capture data signals from the transmitter and send them to a computer using the Ponemah software (Version 5.2, Data Sciences International) to convert the digital output of the receiver into a calibrated analog output. The EEG was recorded continuously (24 h/day, 7 d/week) over a period of 4 weeks.

EEG data were analyzed using NeuroScore (version 3.3.1) software (Data Sciences International) as described previously [22, 23]. Briefly, seizure frequency, seizure duration, and spike numbers were determined using the spike train analysis tool implemented in NeuroScore with the following criteria: threshold value = 7.5 × standard deviation (SD) of the baseline (i.e., activity during artifact- and epileptiform-free epochs) − 1000 μV, spike duration = 0.1–50 ms, spike interval = 0.1–2.5 s, minimum train duration = 30 s, train join interval = 1 s, and a minimum number of spikes = 50. Before spike analysis, the recordings were high-pass filtered at 1 Hz. All EEG recordings were additionally verified by manual screening. Fast Fourier transformation (FFT) was performed to derive absolute *δ* (0.54 Hz), *θ* (4–8 Hz), *α* (8–13 Hz), *β* (13–30 Hz), and *γ* (30–50 Hz) power values during *status epilepticus* (*SE*) and the chronic phase, which were subsequently normalized to baseline activity.

### Immunohistochemistry

For immunohistochemistry, transcardial perfusion was performed using 30 ml PBS (pH 7.4) and 30 ml 4% PFA-containing PBS for fixation. Coronal sections of 40 µm thickness were cut on a cryostat and stored in PBS (pH 7.4) in 24-well plates containing 0.01% sodium azide as a preservative. For immunofluorescence staining, primary antibodies—rabbit anti p-TAK1 (1:100, Cell Signaling # 4536), mouse anti Iba1 (1:300, Millipore, # MABN92), rabbit anti Iba1 (1:400, WAKO # 019–19,741), and mouse anti-S100B (1:200, Abcam # ab16959)—were used. Secondary antibodies Alexa Fluor®594 (1:500, Molecular Probes) and Alexa Fluor®488 (1:500, Molecular Probes) were used. Images were acquired at 1-µm intervals using a confocal microscope (SP8, Leica, Hamburg, Germany). Cell type analysis in the hippocampal CA1 region was performed on image stacks of 290.62 × 290.62 × 20 µm^3^ volumes if not stated otherwise. The images obtained were analyzed using ImageJ software and figures were edited using ImageJ. P-TAK1 fluorescent intensity was measured in the nucleus and for each cell highest mean average value was taken. For Iba1 quantification, the threshold for fluorescent intensity was set at a minimum of 75 and a maximum of 255. The percentage area occupied by Iba1 was measured on all slices of the sections used. For each section, the mean average percentage occupied area from all slices was calculated. For quantification, we used 9 sections from 3 animals for each condition. *p* < 0.05, post hoc Tukey HSD.

### Data Analysis

All error bars in the bar graphs represent SD. The following methods were applied for statistical analysis: Two-sided Student’s *t*-test was used when comparing two groups and variance analysis (ANOVA) followed by Tukey’s posthoc test was used when comparing more than two groups. Two-way split-plot ANOVA was conducted to compare generalized seizure activity over time. In all cases, a significance level of 5% was applied (*p* < 0.05). Box plots represent median and quartiles (25th and 75th percentile) with whiskers extending to the highest and lowest values within 1.5 times the interquartile range.

## Results

### TAK1 Activation in Glial Cells

**Fig. 1:**
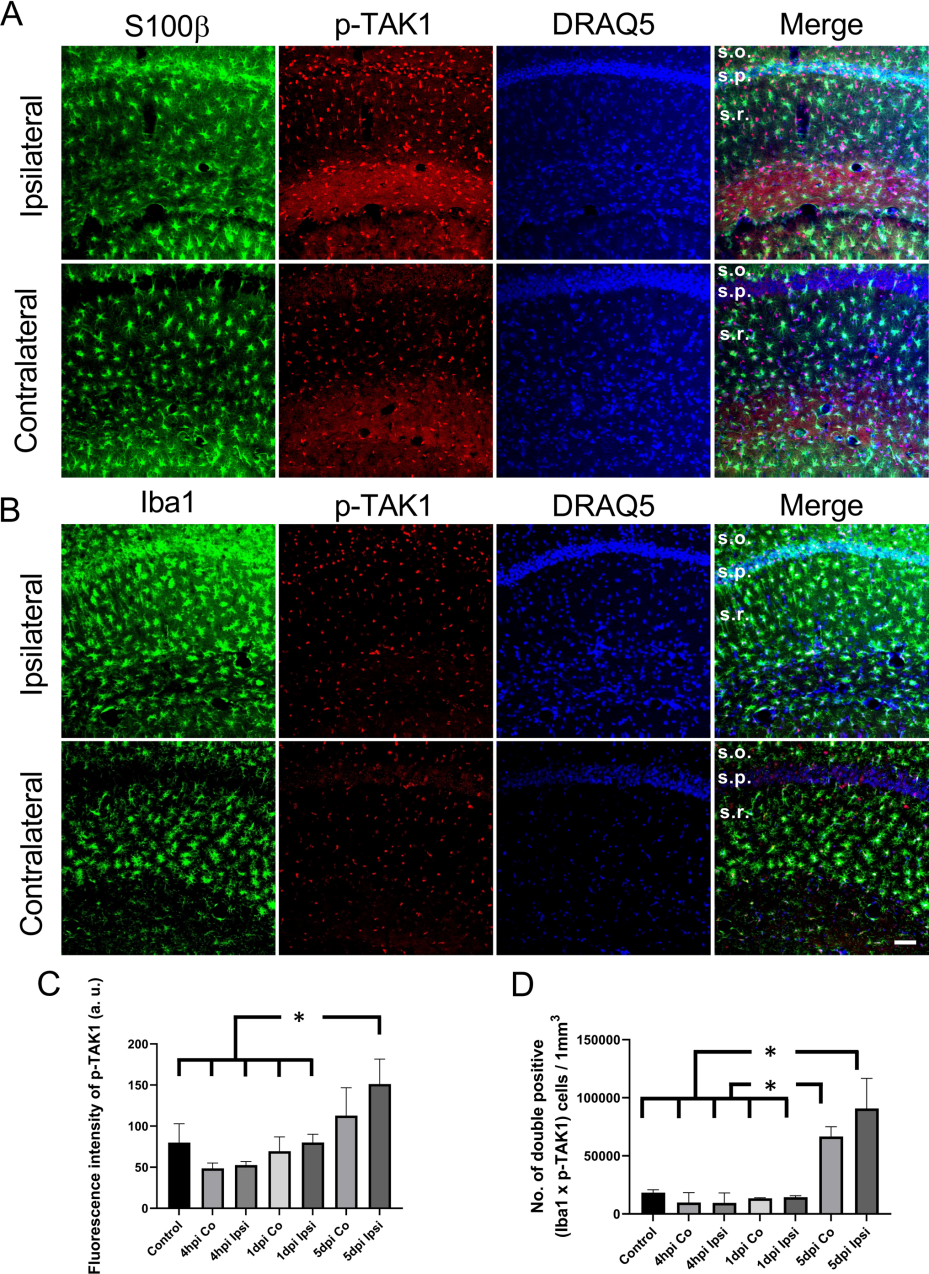
Immunohistochemical characterization of TAK1 activation in glial cells: Slices were prepared from brains of animals perfused 4 h (4 hpi), 1 day (1 dpi) or 5 days (5 dpi) after kainate injection. TAK1 activation was detected by antibodies against the phosphorylated form of the kinase (p-Tak) **A** S100β/p-TAK1 and **B** Iba-1/p-TAK1 double labelling 5 dpi. **A** No colocalization of the two antigens was observed, indicating lack of Tak1 activation in astrocytes at this time point. **B** Double immunostaining showed marked colocalization of p-TAK1 and Iba-1, demonstrating TAK1 activation in microglia 5 dpi. Scale bar, 50 µm. **C** Quantification of the p-TAK1 fluorescence intensity in the nuclei of Iba-1-positive cells at different time points after kainate injection. The fluorescence was significantly increased 5 dpi on the ipsilateral side compared to the untreated control and to the two earlier time points after kainate injection. **D** The number of microglial cells showing TAK1 activation increased 5 dpi on both the ipsi- and the contralateral side. 15 different sections from 3 mice were used in each case except 4 hpi where 10 sections from two mice were used for quantification and cell counting. Analysis was performed in the stratum radiatum of the hippocampal CA1 region (4.5 × 10^5^ µm^3^). *p < 0.05 (ANOVA). s.o. = stratum oriens; s.p. = stratum pyramidale; s.r. = stratum radiatum

To investigate whether Tak1 is activated during early epileptogenesis in microglia and astrocytes, *SE* was induced in C57Bl6 mice by unilateral intracortical kainate injection and immunofluorescence staining of phosphorylated TAK1 in combination with the astrocyte marker S100b and the microglial marker Iba1 was performed at different time points: 4-h post-injection (4 hpi), 1-day post-injection (1 dpi), and 5-day post-injection (5 dpi). TAK1 activation was not observed in S100β-positive astrocytes (Fig. 1A, Suplimentary Fig. 1A). TAK1 activation co-localized with microglial cells (Fig. 1B, Suplimentary Fig. 1B). Fluorescence intensity of p-TAK1 increased significantly on the ipsilateral side 5 dpi when compared to untreated control (Ctr.) and to both ipsi- and contralateral sides of mice at 4 hpi and 1 dpi (fluorescence intensity of p-TAK1: Ctr. = 80 ± 23 a.u. [arbitrary unit]; 4 hpi contra = 48 ± 6 a.u.; 4 hpi ipsi = 52 ± 4 a.u.; 1 dpi contra = 69 ± 17 a.u.; 1 dpi ipsi = 80 ± 9 a.u.; 5 dpi contra = 113 ± 34 a.u.; 5 dpi ipsi = 151 ± 30 a.u.; *p* = 0.0024, ANOVA; Fig. 1C). The number of microglia cells showing TAK1 activity was significantly higher on the ipsi- and contralateral side 5 dpi when compared to the untreated control and to the ipsi- and contralateral sides of mice 4 hpi and 1 dpi (microglia cells/mm^3^ showing TAK1 activity: Ctr. = 18,298 ± 2565; 4 hpi contra = 9773 ± 8543; 4 hpi ipsi = 9618 ± 8381; 1 dpi contra = 13,496 ± 465; 1 dpi ipsi = 14,428 ± 1231; 5 dpi contra = 66,709 ± 8394; 5 dpi ipsi = 90,755 ± 25,908; *p* = 9.1516e-07 Fig. 1D). The present data suggests that TAK1 is activated in microglia in experimental epilepsy.

### Microglia Activation in TAK1 KO Animals

**Fig. 2:**
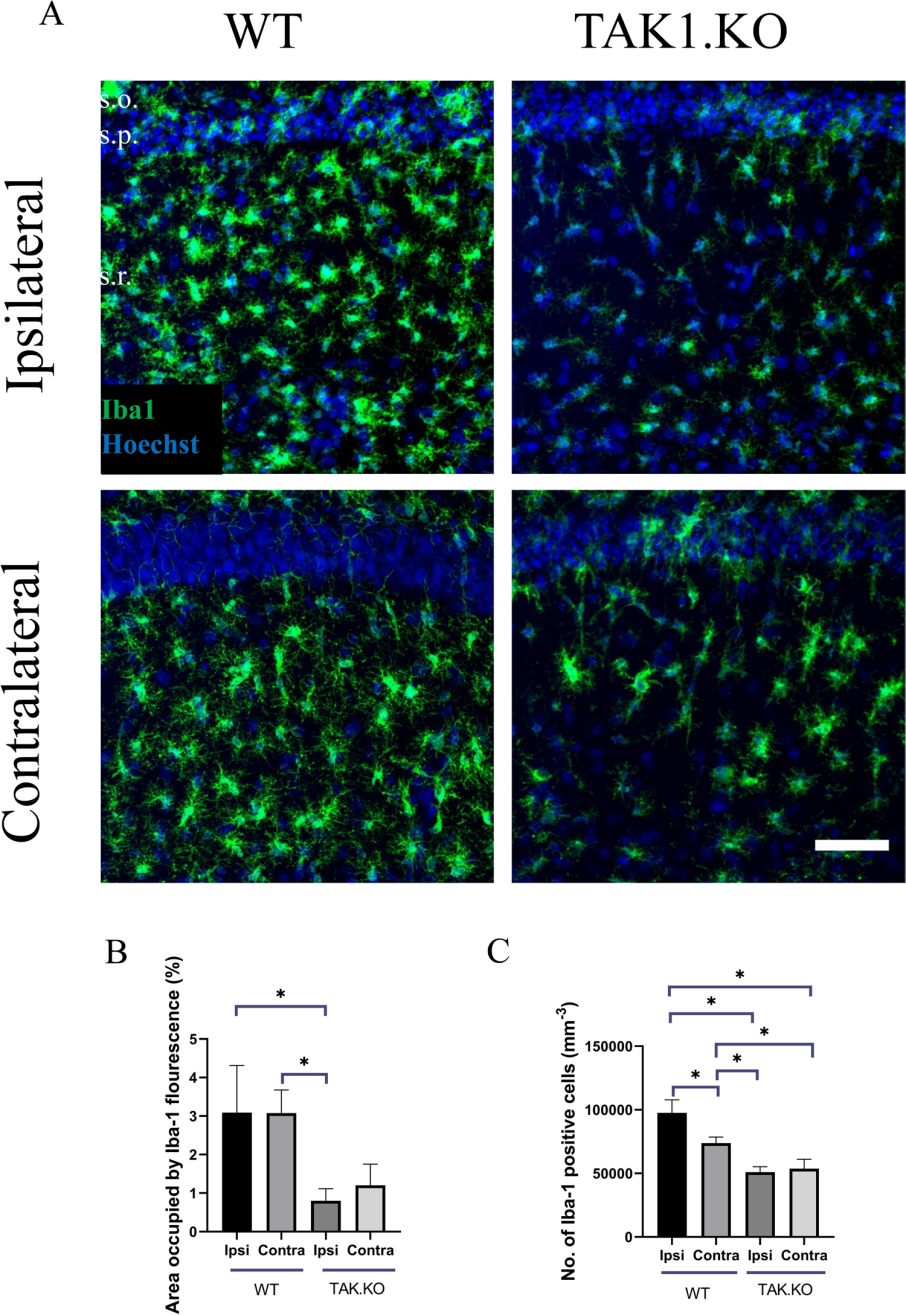
Microglia activation after kainate-induced SE: Slices were prepared from brains of animals perfused 5 dpi. Microglia activation was detected by Iba1 (green)/Hoechst (blue) double staining. **A** Decreased microglial activation was observed in TAK1 KO mice. **B** The area occupied by Iba1-positive cells was significantly lower on the ipsilateral side in TAK1 KO mice and **C** the number of Iba1-positive cells were significantly lower in TAK1 KO animals when compared to their control littermates. Scale bar, 50 µm. *p < 0.05 (ANOVA). s.o. = stratum oriens; s.p. = stratum pyramidale; s.r. = stratum radiatum

Microglia activation was assessed 5 days after *SE* in microglial-specific TAK1 KO and their Cre-negative (control) littermates. Fewer alterations in microglial morphology (hypertrophic cell bodies and processes) were observed in TAK1 KO animals compared to their control littermates (Fig. 2A). Iba1 immunoreactivity was also significantly reduced on the ipsilateral side of TAK1 KO mice than on the ipsilateral and contralateral side of Cre-negative littermates 5 dpi (area occupied by Iba1: TAK1 KO ipsi = 0.802 ± 0.313%; TAK1 KO contra = 1.2 ± 0.55%; control ipsi = 3.089 ± 1.223%; control contra = 3.076 ± 0.6%, *p* = 0.0092, ANOVA; Fig. 2B). The number of Iba1-positive cells observed in a volume of 289 × 289 × 19 µm^3^ was also significantly lower in TAK1 KO mice than in Cre-negative littermates (Iba1-positive cells: TAK1 KO ipsi = 50,902 ± 4244; TAK1 KO contra = 53,703 ± 7397; control ipsi = 97,604 ± 10,258; control contra = 73,868 ± 4679, *p* = 0.0001, ANOVA; Fig. 2C). Because after status epilepticus microglia were activated on both ipsi- and contralateral hemispheres, therefore, both groups ipsi- and contralateral sides have been compared with each other between WT and TAK1 KO animals. Overall, our findings suggest that the number of microglial cells and their degree of reactivity is reduced in the absence of TAK1 in a TLE mouse model.

### Kainate-Induced Acute and Chronic Epileptic Activity in TAK1 KO Animals

**Fig. 3:**
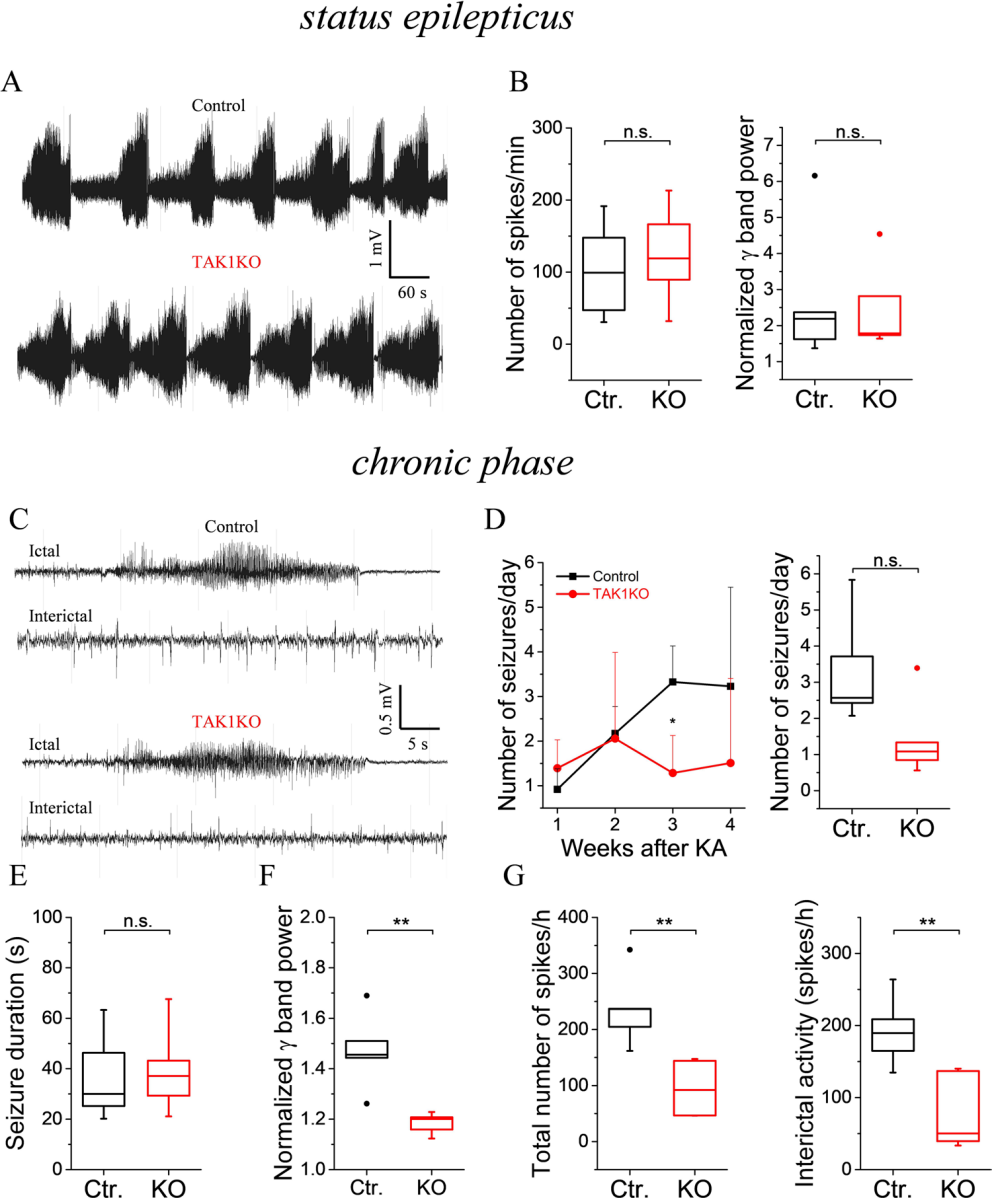
Kainate-induced acute and chronic epileptiform activity in TAK1 KO and control mice. Seizures and interictal activity were detected by continuous telemetric EEG recordings over a period of 4 weeks. **A** Representative EEG traces recorded during SE. **B** Severity of SE in TAK1 KO (KO) and control (Ctr.) mice was quantified by spike frequency and spectral analysis of EEG data recorded during the first 6 h after kainate (KA) injection. Neither the spike activity nor the γ band power was significantly different between the genotypes. **C** Representative EEG traces showing spontaneous generalized seizures (ictal) and interictal spiking activity (interictal) during the chronic phase of epileptogenesis. **D** Average number of *SE*-triggered spontaneous generalized seizures in TAK1 KO and control mice (left graph: week-by-week comparison; right graph: median number during the third and fourth week of recording). Seizure activity was significantly lower in TAK1 KO mice during the third week of recording. **E** The duration of spontaneous generalized seizures was not different between the genotypes. **F** The power of EEG activity in the γ-band range during the third and fourth week of recording was significantly lower in mice lacking microglial TAK1. **G** Spike frequency analysis of the same EEG data showed significantly reduced frequency of total EEG spikes (including spikes during seizures) and interictal EEG spikes in TAK1KO mice compared to control mice. *N* = 5 mice per group. *p < 0.05, **p < 0.01 (two-way ANOVA (left graph in D) and *t*-test). Data in **D** (left graph) represent mean ± SD. Box plots represent median and quartiles (25th and 75th percentile) with whiskers extending to the highest and lowest values within 1.5 times the interquartile range. The dots represent the outliers

To shed light on the role of TAK1 in the development and progression of TLE, TAK1 KO and control mice were subjected to the experimental TLE model, and the epileptic activity during *SE* and the chronic phase was monitored by continuous telemetric EEG recordings over a period of 4 weeks. The severity of KA-induced *SE* was quantified by counting the number of EEG spikes with amplitudes exceeding baseline activity at least 7.5-fold and by assessing the spectral power in the high frequency range after FFT of the EEG data during the first 6 h of recording. The result of the analysis revealed that neither spike frequency (control (median, quartiles): 99.26, 47.1–147.81 vs. TAK1 KO: 119, 89.4–166.4 spikes/min; *p* = 0.64, *t*-test) nor the normalized spectral power (control: 2.2, 1.6–2.4 vs. TAK1 KO: 1.8, 1.7–2.8; *p* = 0.82, *t*-test) were significantly affected by TAK1 deletion during *SE* (Fig. 3A, B). All investigated mice developed chronic spontaneous generalized seizures (Fig. 3C, D). The frequency of seizures during the first two weeks was similar in the experimental groups but significantly lower in TAK1 KO mice in the third week (control: 3.3 ± 0.8 vs. TAK1 KO: 1.3 ± 0.8 seizures/day; *p* = 0.01, two-way ANOVA) (Fig. 3D, left graph). During the fourth week, the seizure frequency in KO mice was less than half compared to controls (control: 3.2 ± 2.2 vs. TAK1 KO: 1.5 ± 1.9 seizures/day), but due to the high variability of seizure activity between individual mice, this difference did not reach statistical significance (*p* = 0.09, two-way ANOVA). The same applies when the average seizures/day during the 3rd and 4th week after epilepsy induction, i.e. the period at which the mice reliably show chronic seizures in this model [21], are considered together (control: 2.6, 2.4–3.7 vs. TAK1 KO: 1.1, 0.8–1.3 seizures/day; *p* = 0.057, *t*-test) (Fig. 3D, right graph). The duration of spontaneous chronic seizures was also not significantly different between the genotypes (control: 30, 25.2–46.3 s vs. TAK1 KO: 37.1, 29.3–43.2 s; *p* = 0.9, *t*-test) (Fig. 3E). The high variability in the number of spontaneous generalized chronic seizures between individual animals in our model hinders statistical analysis and interpretation of the parameter ‘number of seizures/day’. A less variable and therefore more reliable estimate of the severity of epilepsy progression is provided by the spike and power analysis, as they not only consider generalized seizures but the total (i.e. also the interictal) epileptiform activity. Indeed, the normalized power of high-frequency EEG activity during the third and fourth week after KA injection was significantly lower in mice lacking microglial TAK1 than in control mice (control: 1.46, 1.44–1.51 vs. TAK1 KO: 1.2, 1.15–1.21; *p* = 0.004, *t*-test) (Fig. 3F). Likewise, the total number of EEG spikes, as well as the interictal spike activity (determined by subtracting spikes during seizures from total spike activity) were significantly reduced in KO mice (Total spike activity, control: 236, 204–237 vs. TAK1 KO: 92, 46–144 spikes/h; *p* = 0.005, *t*-test; interictal activity, control: 190, 165–209 vs. TAK1 KO: 50, 39–137 spikes/h; *p* = 0.008, *t*-test) (Fig. 3G). Taken together, these data suggest that deletion of microglial TAK1 attenuates chronic epileptic activity without affecting the severity of *SE*.

## Discussion

Here we show that TAK1 activation is absent in astrocytes and present in microglia in experimental TLE. After *SE* an increase in p-TAK1 in the hippocampus has been reported in rats with pilocarpine-induced epilepsy [24]. The increased TAK1 activity after *SE* reported by Tian et al. is not cell-specific [24], while in the current study, we show amplified TAK1 activation in microglia after *SE*. TAK1 deletion in microglia led to reduced microglial activation. Activated microglia release inflammatory cytokines [25, 26], most of which are regulated by NF-κB activation [27]; TAK1 is an upstream modulator of NF-κB [28, 29]. *SE* results in oxidative stress [30], which in turn has been shown to activate TAK1 [31]. Moreover, it has been suggested that hippocampal depolarized neurons can release TNF-α [32], which activates TAK1 [33].

As we observed TAK1 activation predominantly in microglia (Fig. 1B), microglia-specific TAK1 KO mice were used to study its role in epileptogenesis. This mouse line has already been investigated and reported in detail [20, 34]. Microglia are phagocytic descendants of the mesoderm that migrate into the brain during embryonic development [35, 36]. Due to the longevity and self-renewal of microglia, these cells persist throughout the life of the organism without any input from peripheral immune cells [26, 36]. In contrast, peripheral CX3CR1 positive immune cells exhibit much faster turnover rates compared to microglia and are rapidly replaced by CX3CR1 negative bone marrow progenitor cells [20, 37]. Therefore, TAK1 deletion remains restricted to microglia three weeks after TAM administration.

It has already been shown that in experimental autoimmune encephalomyelitis and experimental stroke, microglia-specific TAK1 KO mice showed neuroprotective effects and strongly diminished CNS inflammatory responses including abolished activation of NF-κB and reduced expression or release of inflammatory cytokines (such as IL-1β and TNF-α) and chemokines (such as CCL2) [20, 34]. In the present study, a reduced alteration in microglia morphology (hypertrophic cell bodies and processes) was observed in microglia-specific TAK1 KO mice when compared with their control littermates. Furthermore, TAK1 KO animals showed significantly reduced Iba1 immunoreactivity and a significantly lower number of microglia when compared to the Cre-negative controls. Microglia rapidly respond to noxious stimuli and are activated at a very early stage after an injury or infection [26, 38, 39]. In previous studies, activated microglia have been observed in animal models of epilepsy [9, 11] and in brain tissue obtained from temporal lobe epilepsy (TLE) patients [7, 8]. Microglia activation is characterized by increased proliferation, morphological transformation, and migration to the site of injury [26, 40]. The significantly lower number of microglia in the conditional TAK1 KO mice was presumably due to reduced proliferation or migration (or both) of the cells.

Based on observations from experimental epilepsy and outcomes from clinical studies, it has been suggested that inflammation promotes seizure generation [10, 12, 41–46]. To investigate if the observed reduced inflammatory response in microglia-specific TAK1 KO mice influences seizure frequency in TLE, EEG recordings were performed for 1 month after kainate-induced *SE* in microglial TAK1 KO and control mice. The results showed that mice devoid of microglial TAK1 developed less severe epilepsy following *SE* than their Cre-negative littermates (Fig. 3). The inhibition of TAK1 activation via using its inhibitor 5z-7-oxozeaenol reduced the duration of seizures without effecting spontaneous recurrent seizures in experimental epilepsy in rats [24]. In the present study, the epileptic severity was reduced in TAK1 KO animals without any difference in seizure duration between TAK1 KO mice and their control littermates. This difference in findings between the current study and Tian et al. could be due to all or one of the following reasons: e.g. use of different species, different experimental epilepsy models, and general inhibition of TAK1 by using its inhibitor 5z-7-oxozeaenol vs microglia-specific deletion of TAK1. Tian et al. and our findings suggest that targeting TAK1 can reduce the duration [24] and intensity of seizures in experimental epilepsy. Inflammation and inflammatory cytokines are suggested to promote epileptogenesis [14, 47–49]. Activated microglia have been shown to be the primary source of TNF-α in the mouse model of epilepsy used in the current study [50]. Microglia-specific TAK1 KO mice showed decreased expression and release of IL-1β and TNF-α in CNS in animal models of experimental autoimmune encephalomyelitis and experimental stroke [20, 34]. Also, TAK1 inhibition resulted in reduced IL-1β levels in brain tissue after *SE* [24]. Both IL-1β and TNF-α inhibited astrocyte gap junctions [21, 50], which consequently results in impaired astrocytic K^+^ and glutamate buffering that may lead to neural hyperexcitability and seizure generation [51]. Moreover, IL-1β and TNF-α release from activated microglia inhibit the reuptake of glutamate and promote its release from astrocytes contributing to neuronal excitability [47, 50, 52]. Due to inflammation, the blood–brain barrier (BBB) is disrupted, which allows albumin to enter the brain parenchyma, which consequently results in hyperexcitability via a reduction in glutamate uptake and K^+^ buffering by astrocytes [47]. In addition, a positive correlation between BBB leakage and the frequency of spontaneous seizures has been reported [53]. In microglia-specific TAK1 KO mice, the reduced release of these inflammatory cytokines [20, 34] due to the decreased microglial number and activity (observed in the present study) may result in lower seizure activity.

Taken together, our data suggest that microglial TAK1 activation is involved in the pathogenesis of TLE. Our study has some limitations, such as the small sample size (for EEG 5 animals/group, and for immunostaining 2–3 mice/group were used). Moreover, with current tools, we could not completely dissect the peripheral CX3CR1-positive immune cells that can be replaced in the bone marrow and possibly can invade the epileptic tissue. The data should thus be carefully interpreted for any clinical implications.

## Conclusions

TAK1 is activated in microglia after *SE*. Microglial cells devoid of TAK1 show less activation in mice with kainate-induced epilepsy. Microglial-specific deletion of TAK1 results in reduced epileptic activity in an experimental epilepsy model.

## Supplementary Information

Below is the link to the electronic supplementary material.

Supplementary file1 (DOCX 711 kb)

## Data Availability

All data generated or analyzed during this study are included in this published article.

## Abbreviations

ANOVA: Variance analysis
a.u.: Arbitrary unit
Ctr.: Control
EEG: Electroencephalogram
FFT: Fast Fourier transformation
i.p.: Intraperitoneal
TAK1: TGF-β activated kinase 1
TAM: Tamoxifen
TLE: Temporal lobe epilepsy
4 hpi: 4-H post-injection
1 dpi: 1-Day post-injection
5 dpi: 5-Day post-injection
