# Supplementary material for: TGF-β Activated Kinase 1 (TAK1) Is Activated in Microglia After Experimental Epilepsy and Contributes to Epileptogenesis
Source: Mol Neurobiol. 2023 Mar 2;60(6):3413–22. doi: 10.1007/s12035-023-03290-2 (PMC10122619; doi:10.1007/s12035-023-03290-2)
Supplement: Supplementary file 1 — Supplementary file1 (DOCX 711 kb) [file 12035_2023_3290_MOESM1_ESM.docx]

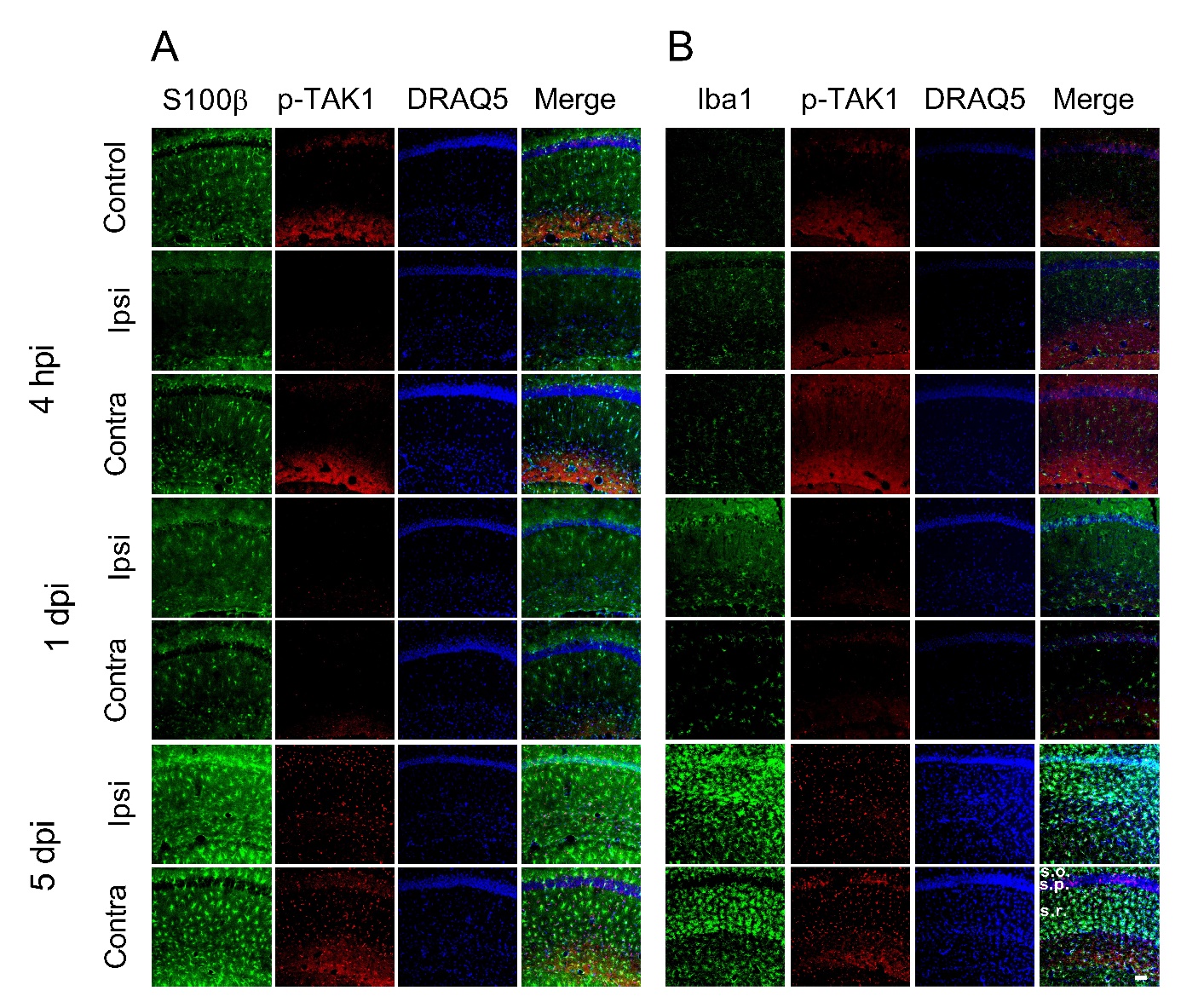


**Supplementary figure. Immunohistochemical characterization of TAK1 activation in glial cells:** Slices were prepared from the brains of animals perfused 4 hours (4 hpi), 1 day (1 dpi) or 5 days (5 dpi) after kainate injection. TAK1 activation was detected by antibodies against the phosphorylated form of the kinase (p-Tak) (A) S100β/p-TAK1 and (B) Iba-1/p-TAK1 double labelling 5 dpi. (A) No colocalization of the two antigens was observed, indicating lack of Tak1 activation in astrocytes at this time point. (B) Double immunostaining showed marked colocalization of p-TAK1 and Iba-1, demonstrating TAK1 activation in microglia 5 dpi. Scale bar, 50 µm. s.o. = stratum oriens; s.p. = stratum pyramidale; s.r. = stratum radiatum.
